# Anomalous Nuclear Overhauser Effects in Carbon-Substituted Aziridines: Scalar Cross-Relaxation of the First Kind**

**DOI:** 10.1002/anie.201410271

**Published:** 2015-01-28

**Authors:** Ilya Kuprov, David M Hodgson, Johannes Kloesges, Christopher I Pearson, Barbara Odell, Timothy D W Claridge

**Affiliations:** Department of Chemistry, University of Oxford, Chemistry Research Laboratory12 Mansfield Road, Oxford OX1 3TA (UK); School of Chemistry, University of Southampton, Highfield CampusSouthampton SO17 1BJ (UK)

**Keywords:** aziridines, NMR spectroscopy, NMR theory, Overhauser effect, scalar relaxation

## Abstract

Anomalous NOESY cross-peaks that cannot be explained by dipolar cross-relaxation or chemical exchange are described for carbon-substituted aziridines. The origin of these is identified as scalar cross-relaxation of the first kind, as demonstrated by a complete theoretical description of this relaxation process and by computational simulation of the NOESY spectra. It is shown that this process relies on the stochastic modulation of *J*-coupling by conformational transitions, which in the case of aziridines arise from inversion at the nitrogen center. The observation of scalar cross-relaxation between protons does not appear to have been previously reported for NOESY spectra. Conventional analysis would have assigned the cross-peaks as being indicative of a chemical exchange process occurring between correlated spins, were it not for the fact that the pairs of nuclei displaying them cannot undergo such exchange.

Among the powerful NMR structure and conformation elucidation techniques,[1] nuclear Overhauser effect (NOE) experiments play a central role because they map the spatial proximity of neighboring spins.[2] The experimental techniques used to observe magnetization transport due to NOEs (NOE spectroscopy or NOESY) can also reveal the presence of chemical exchange processes, for which the methods are also known as exchange spectroscopy (EXSY).

For small molecules (*M*_r_< ca. 1000 Da) in non-viscous liquids at ambient temperatures, ^1^H-^1^H cross-peaks are negative (opposite sign to the diagonal-peaks) for the NOE and positive (same sign as the diagonal-peaks) for pairs of signals undergoing chemical exchange. For large molecules (*M*_r_> ca. 2000 Da), aggregates or with viscous solvents, the sign of the ^1^H-^1^H Overhauser effect undergoes a well-documented inversion and cross-peaks become positive, appearing similar to those arising from chemical exchange processes.

The theory of spin relaxation processes in general[3] and of NOEs in particular[2] is one of the most developed areas of magnetic resonance spectroscopy, meaning deviations from the behaviors described above are rare indeed. Herein, we report anomalous NOESY observations for a series of C-substituted NH aziridines **1 a**–**d**, for which the usual interpretations offered no acceptable explanation.

During the development of methodology for the synthesis and desulfinylation of *N*-sulfinyl terminal aziridines by some of the current authors,[4] extensive spectroscopic analysis was carried out to establish the integrity of the aryl-substituted NH aziridines **1 a,b** (due to prior literature mischaracterization[5]).


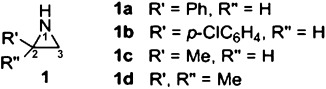


A NOESY spectrum of 2-phenylaziridine (**1 a**) in dry CD_2_Cl_2_ at 298 K exhibited unexpected strong positive cross-peaks between the broad N-H resonance and all three CH protons of the aziridine (Figure 1 a), in addition to the anticipated NOEs producing negative cross-peaks between aziridine CH protons, characteristic of rapidly tumbling molecules. The aziridine CH protons also produced the expected negative cross-peaks with the nearby phenyl protons. When the temperature was reduced to 193 K, the anomaly disappeared and only the anticipated patterns of negative (spatial proximity) and positive (chemical exchange) cross-peaks were apparent (Figure 1 b).

**Figure 1:**
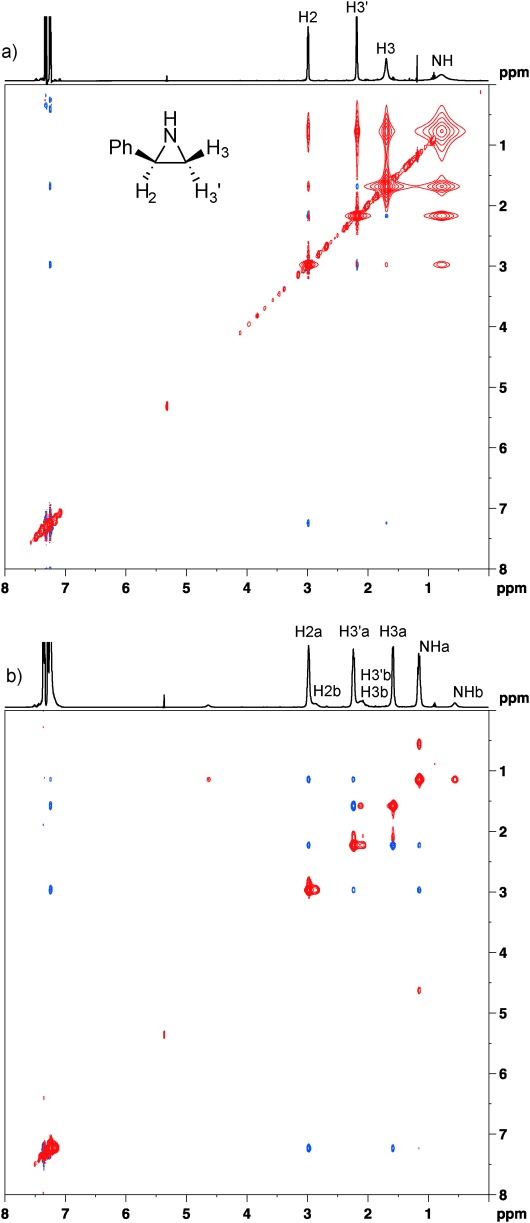
2D ^1^H NOESY spectra recorded at 500 MHz with 800 ms mixing times for 2-phenylaziridine (1 a) in CD_2_Cl_2_ at a) 298 and b) 193 K. At 298 K the two invertomers exhibit fast exchange and anomalous cross-peaks appear between the NH and its scalar coupled partners (red), in addition to dipolar NOE cross-peaks (blue). At 193 K the invertomers are in slow exchange and the expected exchange cross-peaks may be observed between these species (red) in addition to dipolar NOE cross-peaks (blue).

The exchange process observed is well understood—slow inversion at the nitrogen center is a known feature of aziridines,[6] and at low temperature this process gave rise to two conformational forms (nitrogen invertomers), in a 5:1 ratio (see Figure S1 in the Supporting Information), with the dominant isomer having the NH and phenyl group *trans* to each other, as demonstrated by NOE analysis.

While the low-temperature NOESY spectrum can be readily rationalized, the spectrum at 298 K is clearly anomalous; although the higher temperature accelerates the conformational interconversion process between the invertomers, there can be no chemical exchange of any kind between the CH and NH protons linked by the positive cross-peaks. The molecule is small and the solvent non-viscous, meaning that the Overhauser effect must produce negative cross-peaks; indeed these are apparent in Figure 1 a and are consistent with the aziridine structure. The *p*-chlorophenyl aziridine (**1 b**) showed similar behavior[7] and further investigations revealed the phenomenon is not restricted to aryl-substituted NH aziridines, being also observed with 2-methylaziridine (**1 c**) and 2,2-dimethylaziridine (**1 d**; see Figures S2–S5), prompting us to seek a rational explanation for this behavior.

A variety of mechanisms were considered but ruled out, experimentally and/or theoretically. The most obvious process of direct chemical exchange could be discounted, as there is no chemically feasible process by which the NH and CH protons could exchange. As evidence, samples in which the NH proton was chemically exchanged for deuterium (by the addition of D_2_O) showed no sign of loss of CH resonance intensity on long-term storage. It is possible for relayed NOEs to have the opposite sign to direct NOEs in small molecules,[1,2] but this mechanism was also rejected because available pathways for these effects were not consistent with the anomalous patterns observed and because the anomalous peaks were often of greater intensity than the NOEs present. Further possibilities considered and rejected included quadrupolar effects involving ^14^N (^15^N-labelled **1 a** exhibited the same phenomenon; Figure S6), all cross-correlation mechanisms between quadrupole, dipole–dipole and chemical shift anisotropy interactions, and the modulation of interproton distances by conformational exchange.[7]

After elimination of these mechanisms, the one remaining candidate was the stochastic modulation of *J*-couplings taking place during nitrogen center inversion. This inversion process would alter the HN-CH dihedral angles, leading to a variation in the magnitude of the associated vicinal ^3^*J*_NH-H_ values. This could, in principle, cause cross-relaxation between the *J*-coupled spins through the process known as scalar relaxation of the first kind (SRFK)[2b] which, as detailed below, proved to be the mechanism underlying the anomalous observations. We believe this to be the first reported observation of scalar cross-relaxation detected in 2D NOE spectra. Since this is a little known phenomenon in mainstream NMR spectroscopy, it is pertinent to expand on the details of this mechanism and we herein provide a complete theoretical rationalization and computational simulation for this process.

Following Redfield,[3a] we split the Hamiltonian of a two-spin system into the static part 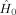 and the centered stochastic part 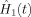 [Eq. (1)].

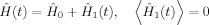
(1)

Then, for the case of stochastically modulated *J*-coupling, one obtains Equation (2),

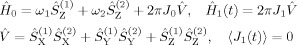
(2)

where angular brackets denote ensemble average, *ω*_1,2_ are Zeeman frequencies of the two spins, 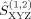 are spin operators, *J*_0_ is the static part of the *J*-coupling and *J*_1_(*t*) is its time dependent part. Formal application of Bloch-Redfield-Wangsness relaxation theory[3a] then leads to the following expression for the relaxation superoperator given in Equation (3).

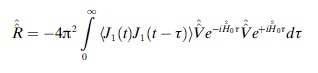
(3)

Assuming an exponential correlation function for the stochastic part of the *J*-coupling [Eq. (4)],

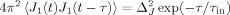
(4)

the automatic symbolic processing system developed by Kuprov et al.[8] returned the expression defining the “scalar cross-relaxation” rate between the two spins [Eq. (5)],

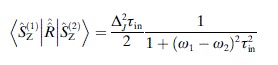
(5)

where *τ*_in_ is the correlation time of the nitrogen inversion process and Δ_*J*_ is the modulation depth of the scalar coupling. The difficulty with this expression is that 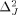 is tiny—about 15 Hz variation in the *J-*coupling was estimated from a DFT calculation on 2-methylaziridine (Figure 2)—and therefore cannot be expected to compete with dipole–dipole (DD) cross-relaxation process with its 10 kHz modulation depth (for computational simplicity 2-methylaziridine (**1 c**) was used; *J* values could not be measured experimentally, even at low temperatures, due to a lack of resolved coupling structure). The caveat is that the correlation time *τ*_in_ of the nitrogen center inversion in aziridines is seven orders of magnitude longer than the rotational correlation time *τ*_R_, meaning the contribution from scalar cross-relaxation becomes significant.

**Figure 2:**
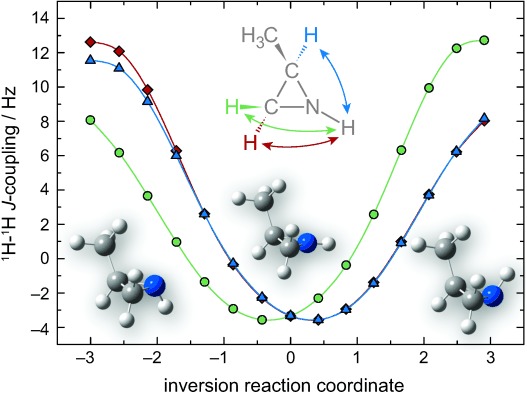
Modulation of three-bond ^1^H-^1^H *J*-couplings to the amine proton during aziridine nitrogen center inversion. The minimum-energy reaction path was obtained for 2-methylaziridine (1 c) with STQN DFT M06/cc-pVTZ method[10] in Gaussian09 with solvent effects accounted for using SCIPCM model.[11] *J*-couplings were then computed for each point on the reaction path using GIAO DFT M06/cc-pVTZ method with IEFPCM solvent model.[12] The Fermi contact contribution to the *J*-coupling was evaluated with a decontracted basis set augmented with tight basis functions.[13] The points in the Figure are interpolated with a cubic spline. See Figure S7 in the Supporting Information for the modulation graphs for *J*-couplings not involving the amino proton.

In liquid-state NMR systems, both the dipole-dipole cross-relaxation and the scalar cross-relaxation of the first kind derived above are very well researched individually.[9] The salient point here is that for small values of the rotational correlation time *τ*_R_ (i.e. for small molecules in non-viscous liquids), the corresponding rates have opposite signs [Eq. (6)],

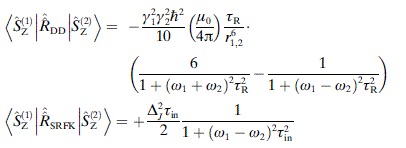
(6)

where *γ*_1,2_ are magnetogyric ratios of the two nuclei and *r*_1,2_ is the internuclear distance. Thus, as illustrated below, it is possible for these two processes to compete with each other. Under appropriate experimental conditions it is also possible for the SRFK term to dominate the dipolar term and produce the unexpected cross-peak sign inversion described above.

All parameters required by the formalism presented above are either known or could be estimated with sufficient accuracy from electronic structure theory calculations,[7] and the *J*-coupling modulation depth was estimated at 15 Hz (Figure 2).

Figure 3 shows the derived cross-relaxation rates for the dipolar process (blue curve), and scalar process (red curve) between the amine proton and a vicinal CH proton in 2-methylaziridine (**1 c**). It is clear that, while the purely dipolar process gives the expected negative cross-relaxation rate (negative cross-peaks in NOESY), the apparent cross-relaxation rate between the protons with modulated J-couplings is predicted to be positive (positive cross-peaks in NOESY). Also positive would be the cross-peaks correlating nuclei that undergo chemical exchange during the nitrogen ring-inversion process.

**Figure 3:**
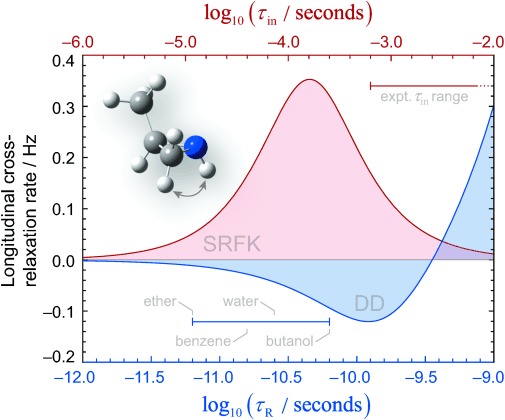
Dipolar (blue curve) and SRFK (red curve) longitudinal cross-relaxation rates between the amine proton and the CH_2_ proton *trans* to the methyl group in 2-methylaziridine (1 c), as functions of rotational correlation time *τ_R_* and nitrogen inversion correlation time *τ*_in_. The curves were obtained from Equation (6) with the following parameters: NMR magnet induction of 11.75 Tesla, proton Zeeman frequency difference of 1000 Hz, interproton distance of 2.33 Å, *J*-coupling modulation depth of 15 Hz. The experimentally reported[14] range of times *τ*_in_ characteristic of the inversion process in aziridines is shown with a red line in the upper part of the graph. The rotational correlation times *τ_R_* of 2-methylaziridine (1 c) in four solvents shown in the lower part of the graph were estimated using the Stokes–Einstein equation, for which the molecular volume of 108 Å^3^ was computed as the PCM DFT solvent-excluded volume.[12] Room-temperature dynamic viscosities of 0.224, 0.604, 0.890, and 2.544 MPa s were used for ether, benzene, water, and 1-butanol, respectively.

A simulation, shown in Figure 4, incorporating the SRFK contribution to the relaxation superoperator and the nitrogen ring-inversion contribution to the chemical kinetics superoperator, using the standard functionality available in Spinach,[15] was capable of reproducing simultaneously the regular-looking NOESY (Figure 1 b) and the anomalous NOESY (Figure 1 a) for 2-phenylaziridine (**1 a**). The sign of every single cross-peak is correctly reproduced, and the role played by the SRFK process is essential in reproducing the anomalous cross-peaks—when SRFK terms are removed from the relaxation superoperator, the agreement with the experiment disappears (Figure S8; the simulation source code is a part of the example set included with Spinach library versions 1.5 and later). The SRFK process has no dependence on inter-nuclear distance, but requires that the scalar coupling between two spins is modulated at a rate that is comparable to the frequency difference between the coupled spins, and that the coupling constant has significant magnitude relative to the shift difference. These factors have meant that scalar relaxation effects have been considered unlikely to be apparent in NOE experiments performed with modern high-field spectrometers.[16]

**Figure 4:**
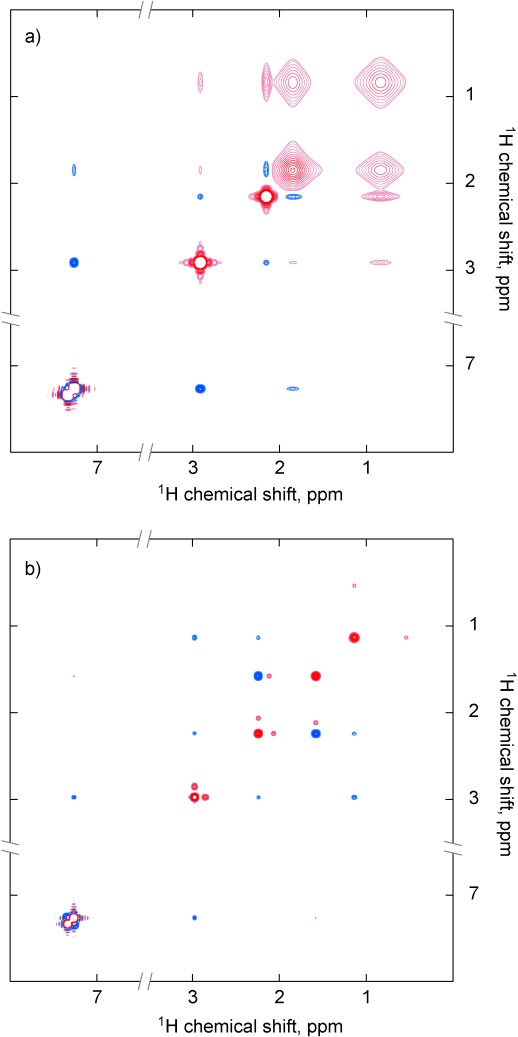
Theoretical ^1^H NOESY spectra of 2-phenylaziridine (1 a), simulated using Spinach library,[15] with the conditions matching the experimental conditions reported in Figure 1, magnetic interaction parameters computed as described for 2-methylaziridine (1 c) in the caption to Figure 3 and the following additional assumptions: for a) forward nitrogen inversion rate constant 1.2 kHz, backward nitrogen inversion rate constant 1.2 kHz, rotational correlation time 50 ps; for b) forward nitrogen inversion rate constant 4 Hz, backward nitrogen inversion rate constant 20 Hz, rotational correlation time 50 ps.

We believe scalar cross-relaxation of the first kind has not previously been reported as being observed in 2D NOESY spectra and, it appears, the effect has only rarely been detected experimentally. The clearest example of this appears to be the work of Fukumi et al.[17] over forty years ago in which negative NOEs were observed between the coupled CH-OH protons of methanol and ethanol and attributed to scalar relaxation. The effect occurred only at acidic pH where the hydroxyl proton exchange rates fulfilled the SRFK requirements, outside which only conventional dipolar NOEs were detected.

We are also not aware of the scalar cross-relaxation process being previously reported for nitrogen NH groups. In the case of the aziridines, as noted earlier, it is well-documented that inversion of the pyramidal nitrogen center is a relatively slow process,[6] and it appears that the reported rates for this process are appropriate to induce the observed SRFK effects detailed herein.[14] In a relevant NMR study of H-N inversion in 2,2-dimethylaziridine (**1 d**),[18] the unimolecular inversion mechanism gave an inversion rate constant of 1.6 s^−1^ at 304 K and an activation free energy at this temperature of 73 kJ mol^−1^. This slow inversion rate when compared to other amines is attributed to the small 60° C-N-C angle (*θ*) at the inverting center which destabilizes the planar transition state relative to the pyramidal nitrogen.[6b] By comparison, the inversion barrier for ammonia is significantly lower at 24 kJ mol^−1^,[19] for pyrrolidines, where *θ*=105°, it’s a comparable 25 kJ mol^−1^ and for azetidines, where *θ*≈90°, the barrier rises slightly to 30 kJ mol^−1^.[20] We note that in control experiments with azetidine and pyrrolidine the anomalous cross-peaks were not observed (Figure S9).

Although the intramolecular inversion process is therefore a likely and appropriate mechanism to modulate the *J*-couplings of the NH-CH protons, we cannot completely exclude the role of intermolecular NH exchange in these systems that would also lead to similar inversion and *J*-modulation, since bimolecular NH exchange between aziridine molecules can occur[18]—a process which may also be catalyzed by water. Indeed, NH exchange with residual water is sometimes observed in NOESY spectra of the aziridines, although its presence was not a requirement for the anomalous cross-peaks to be observed as these were also seen in samples subjected to drying conditions (Figures S2–S6). Nonetheless, the salient point we seek to highlight here is that the anomalous cross-peaks observed in the NOESY spectra of aziridine derivatives owe their presence to scalar cross-relaxation of the first kind. Through a specific combination of parameters, the resulting cross-relaxation process becomes strong enough to overpower the dipolar NOE and invert the sign of the NOESY cross-peak. This phenomenon is likely to be observed in any NMR system where homonuclear *J*-couplings are modulated on a millisecond time scale. We anticipate others will recognize these effects in small molecule NOESY data, now that these apparently anomalous peaks have been reported and rationalized; other similar results from our laboratory will be reported in due course.
