# Supplementary material for: Anomalous Nuclear Overhauser Effects in Carbon-Substituted Aziridines: Scalar Cross-Relaxation of the First Kind
Source: Angew Chem Int Ed Engl. 2015 Jan 28;54(12):3697–701. doi: 10.1002/anie.201410271 (PMC4506550; doi:10.1002/anie.201410271)
Supplement: Supplementary file 1 [file anie0054-3697-sd1.pdf]

Angewandte Chemie
Eine Zeitschrift der Gesellschaft Deutscher Chemiker

Supporting Information

Anomalous Nuclear Overhauser Effects in Carbon-Substituted Aziridines: Scalar Cross-Relaxation of the First Kind**

*Ilya Kuprov, David M. Hodgson, Johannes Kloesges, Christopher I. Pearson, Barbara Odell, and Timothy D. W. Claridge**

anie_201410271_sm_miscellaneous_information.pdf

Contents

Materials and methods

Summary of unsuccessful relaxation theory attempts

Figure S1: ^{1}H NMR Spectra of phenylaziridine (**1a**) at 298 K and 193 K (CD_2Cl_2, 500 MHz)

Figure S2: NOESY Spectrum of 2-phenylaziridine (**1a**) in CD_2Cl_2 at 298 K (500 MHz)

Figure S3: NOESY Spectrum of 2-(chlorophenyl)aziridine (**1b**) in CD_2Cl_2 at 298 K (500 MHz)

Figure S4: NOESY Spectrum of 2-methylaziridine (**1c**) in CD_2Cl_2 at 298 K (500 MHz)

Figure S5: NOESY Spectrum of 2,2-dimethylaziridine (**1d**) in CD_2Cl_2 at 298 K (500 MHz)

Figure S6: NOESY spectrum of the isotopically enriched ^{15}N-2-phenylaziridine (**1a**) in CD_2Cl_2 at 298 K (500 MHz)

Figure S7: Modulation of vicinal and geminal ^{1}H-^{1}H *J*-couplings not involving the amine proton during aziridine nitrogen centre inversion process.

Figure S8: Theoretical NOESY Spectra of 2-methylaziridine (**1c**) with and without SRFK terms included

Figure S9: NOESY Spectra of azetidine (**2**) and pyrrolidine (**3**) in $CDCl_3$ at 298 K (500 MHz)

References

Materials and methods

Synthesis

*2-Phenylaziridine (**1a**)*[1]

2-Amino-2-phenylethanol (9.60 g, 70 mmol) was dissolved in Et_2O with gentle warming and the resulting solution treated with gaseous HCl until pH 1 was reached. After stirring for 5 min, the white precipitate was collected by filtration and washed with Et_2O (3 x 50 mL). The solid was suspended in $CHCl_3$ (150 mL) before addition of $POCl_3$ (22.9 g, 150 mmol) and the reaction mixture then heated to reflux for 18 h; during this time the white suspension turned into a yellow solution. The reaction mixture was then evaporated under reduced pressure, and the resulting yellow oil taken up in H_2O (60 mL) and filtered. THF (60 mL) and solid KOH were

added to the filtrate until pH 12-14 was reached, and the resulting biphasic mixture was then heated to 50 ^{o}C overnight. After cooling to room temperature, addition of Et_2O (20 mL) and partitioning, the aqueous layer was extracted with Et_2O (20 mL) and EtOAc (2 x 20 mL). The combined organic layers were washed with H_2O (30 mL) and brine (30 mL), dried (Na_2SO_4) and concentrated under reduced pressure. Purification (Si-gel, Et_2O with 3% Et_3N) of the residue gave 2-phenylaziridine (**1a**) as a pale yellow oil (5.12 g, 43%). ^{1}H and ^{13}C NMR data as lit.[2]

^{15}N-2-Phenylaziridine[3]

(2-Bromo-1-phenylethyl)dimethylsulfonium bromide[3] (763 mg, 2.34 mmol) was added in one portion to a stirring solution of $^{15}NH_4Cl$ (500 mg, 9.35 mmol) in NaOH (2 M, 4.68 mL, 9.35 mmol). After 22 h, the reaction mixture was extracted with Et_2O (3 x 5 mL), and the combined organic layers dried (Na_2SO_4) and concentrated under reduced pressure. Purification (Si-gel, EtOAc/petroleum ether, 4:1→1:0) of the residue gave ^{15}N-2-phenylaziridine as a colorless oil (42 mg, 15%).

*2-(4-Chlorophenyl)aziridine (**1b**)*

H_2O (15 mL) and KOH (0.28 g, 25 mmol) were added to a solution of 2-(4-chlorophenyl)-2-chloroethylamine hydrochloride[4] (1.13 g, 5 mmol) in THF (15 mL) and the reaction mixture then heated to 50 ^{o}C for 6 h. After cooling to room temperature, addition of Et_2O (15 mL) and partitioning, the aqueous layer was extracted with EtOAc (3 x 10 mL). The combined organic layers were washed with H_2O (20 mL) and brine (20 mL), dried (Na_2SO_4) and concentrated under reduced pressure. Purification (Si-gel, petroleum ether/Et_2O, 1:2) of the residue gave 2-(4-chlorophenyl)aziridine (**1b**) as a yellow oil, which solidified upon standing (0.46 g, 60%). mp 44-45 ^{o}C, lit.[5] 40-42 ^{o}C; ^{1}H and ^{13}C NMR data as lit.[6]

*2-Methylaziridine (**1c**)*

2-Methylaziridine (**1c**) was obtained commercially (Aldrich) and distilled from KOH[7] prior to NMR analysis.

*2,2-Dimethylaziridine (**1d**)*

2,2-Dimethylaziridine[8] (**1d**) was distilled, saturated with KOH and decanted prior to NMR analysis.

NMR Spectroscopy

NMR data were collected on Bruker AVANCE AVII500 or DRX500 spectrometers with sample temperatures over the range 193-298 K as stated. Samples were dissolved in dry C_6D_6, CD_2Cl_2, DMSO-d_6 or toluene-d_8 and characterized by ^{1}H, COSY, edited-HSQC and ^{13}C NMR. 2D NOESY spectra were collected using the standard Bruker pulse programs *noesyph* or *noesygpph* (90-t_1-90-τ_m-90-Acq, without or with a purging bipolar gradient pulse pair (G_1-180-[-]G_1) at the midpoint of the mixing period τ_m, respectively) with mixing times of 800 ms at the temperatures and in the solvents stated. For each data set, 8 or 16 transients were acquired for each of 256 increments with 2K data points per spectrum, using recovery times of 2s. Data were processed typically as 1K x 1K data points using shifted squared-sinebell apodisation windows (WDW=QSINE, SSB=2). Typical sample concentrations for NOE analyses were in the range 15-170 mM, with a maximum of 650 mM. No concentration dependent effects on ^{1}H spectra were seen indicating sample aggregation was not present, and the SRFK peaks were observed at all concentrations studied.

Spin dynamics simulations

For the numerical simulation of cross-relaxation processes, all parameters required were either known or could be estimated with sufficient accuracy from electronic structure theory calculations. Inter-proton distances were between 2.3 and 2.9 Å (from DFT M06/cc-pVTZ geometry optimization), rotational correlation time was estimated to be 15 ps (from PCM DFT M06/cc-pVTZ solvent-excluded volume of 108 Å^3 and Stokes-Einstein equation with $\eta = 6.04\cdot10^{-4}$ Pa·s at T = 298 K), the nitrogen center inversion correlation time was between 10^{-3} and 10^{-1} s (from experimental exchange rate measurements[9]), and the J-coupling modulation depth was estimated to be 15 Hz (Figure 2 of the main text).

The calculation of NOESY spectra was performed using *Spinach*[10] library version 1.5 using IK-1(5,3) basis set and Redfield relaxation theory with correlation time parameters as given in the main text. 2D spectra were produced using explicit time-domain simulation in Liouville space.[10-11] Inter-nuclear dipolar couplings were extracted from the atomic coordinates at the DFT energy minimum geometry obtained as described in the main text. ^{14}N quadrupolar couplings were obtained from the electric field gradients computed at the energy minimum geometry using DFT M06/cc-pVTZ method.[12] Chemical shift tensors were obtained using GIAO DFT M06/cc-pVTZ method and their isotropic parts were replaced with the experimentally measured chemical shifts. The complete simulation code, along with the Spinach environment necessary to run it, is included into the example set of Spinach version 1.5 and later (250+ MB, hence not included here).

Summary of unsuccessful relaxation theory attempts

The various relaxation pathways considered in the course of our investigation that potentially could have been responsible for the anomalous cross-peaks observed in the NOESY spectra are described here. Given that the effect was associated with the NH resonance, quadrupolar effects involving ^{14}N were initially considered. However, a brute-force *Spinach*[10] calculation of NOESY spectra with the quadrupolar relaxation superoperator (including all cross-correlations) added to the Liouvillian alongside the dipolar (DD) and chemical shift anisotropy (CSA) relaxation contributions did not produce the experimentally observed cross-peak pattern. This also applied to indirect quadrupolar effects, such as scalar relaxation of the second kind (SRSK) arising from ^{1}H-^{14}N spin coupling,[13] which are accounted for by the *Spinach* relaxation theory module. In those calculations SRSK was found to be responsible, as it usually is,[13] only for the broadening of the NH proton resonance. Furthermore, experimentally, the ^{15}N-labelled 2-phenylaziridine was found to exhibit the same anomalous cross-peak behavior as observed for the natural abundance substances (Figure S6), ruling out any possible role for ^{14}N quadrupolar coupling.

In another attempt at explaining the NOESY cross-peak anomalies, all types of rotational cross-correlation[14] between DD, CSA and NQI interaction tensors were considered. They are fully accounted for by the relaxation theory module of *Spinach.*[15] Nevertheless, for reasonable values of anisotropies and orientations of the interaction tensors (estimated using DFT calculations as described in *Materials and methods* above), as well as for the expected range of rotational correlation times, no selective cross-peak inversion of the kind shown in Figure 1a of the main text was observed in the simulated spectra. The usual global inversion of the NOE[16] with increasing rotational correlation time was observed, but for all cross-peaks at once and only with the rotational correlation time two orders of magnitude greater (nanoseconds) than that expected for standard organic solvent solutions of methyl- and phenylaziridine (below 100 picoseconds).

Translational contributions to dipolar cross-relaxation processes presented another possible mechanism. Nitrogen center inversion in aziridines is accompanied by significant changes in distances between protons and the resulting exchange modulation of the scalar multiplier in front of the dipolar interaction tensor could in principle play a role in the cross-relaxation behavior. The relaxation effects of the translational modulation of dipolar interaction are well researched,[17] but the analytical expressions resulting from the treatment of simultaneous translational and rotational modulations[18] are cumbersome and restrictive in their assumptions. Sufficient computing power being available, we proceeded to treat the problem in a purely numerical brute-force way – a random walk within a spherical layer was generated with user-specified distance ranges, rotational and translational diffusion coefficients and the Bloch-Redfield-Wangsness theory integral[11b, 19] for a two-spin system was then taken numerically:

$$\hat{\hat{R}} = -\int_0^\infty \left\langle \hat{\hat{H}}_1(t) e^{-i\hat{\hat{H}}_0\tau} \hat{\hat{H}}_1(t-\tau) e^{+i\hat{\hat{H}}_0\tau} \right\rangle d\tau, \qquad \hat{H}_0 = \omega_1 \hat{S}_Z^{(1)} + \omega_2 \hat{S}_Z^{(2)}$$
$$\hat{H}_1(t) = -\frac{\mu_0}{4\pi} \frac{\gamma_1\gamma_2\hbar}{r(t)^5} \left[3\left(\hat{\vec{S}}_1 \cdot \vec{r}(t)\right)\left(\vec{r}(t) \cdot \hat{\vec{S}}_2\right) - r(t)^2 \left(\hat{\vec{S}}_1 \cdot \hat{\vec{S}}_2\right) \right] \qquad (1)$$

In this equation double hats on the Hamiltonians denote commutation superoperators, $\vec{r}\left(t\right)$ is the distance vector between the spins, γ_1 and γ_2 are magnetogyric ratios, $\hat{\vec{S}}_1$ and $\hat{\vec{S}}_2$ are Cartesian vectors of spin operators and $\omega_{1,2}$ are Zeeman frequencies. A more detailed description of the Bloch-Redfield-Wangsness theory module implemented in the *Spinach* library and used here for computing $\hat{\hat{R}}$ may be found in a recent paper by Kuprov *et al*.[15a] An examination of the resulting relaxation superoperator for a realistic ranges of hydrodynamic radii and solvent viscosities did not reveal any anomalous behavior of the kind seen in Figure 1a – the sign of all inter-proton NOE effects did change when the rotational correlation time crossed the usual 0.5 ns threshold (this is when the "classical" NOE changes sign), but not for the picosecond-range rotational correlation times expected for standard organic solvent solutions of methyl- and phenylaziridine. Because the inter-nuclear dipolar interaction is traceless, slowing down the internal motion does not help – the overall correlation function under the integral in Equation (1) is still damped at the picosecond-scale rotational rates and the longitudinal cross-relaxation rates remains negative. Therefore, the inter-proton distance modulation of dipolar effects is not responsible for the observed NOESY cross-peak anomalies.

Having ruled out the above effects, we proceeded to consider scalar relaxation of the first kind, which yielded the successful description presented in the main text that fully accounts for all observed phenomena.

^{1}H and 2D NOESY spectra of Aziridines 1(a-d) and related compounds

Figure S1: ^{1}H NMR Spectra of phenylaziridine (**1a**) at 298 K and 193 K (CD_2Cl_2, 500 MHz). At low temperature two conformational species can be observed (invertomers) in a ~ 5:1 ratio due to the slow inversion of the nitrogen center. Only at the higher temperature do scalar relaxation cross peaks appear in the NOESY spectrum. The *cis-trans* stereochemistry of the two invertomers and the definition of H3 and H3' were established from NOE studies at low temperature. The substantially broader H3 resonance at 298 K occurs because the two protons of the invertomers are only just beyond their coalescence point at this temperature, while H2 and H3' display fast exchange behavior due to smaller shift differences between the two invertomers.

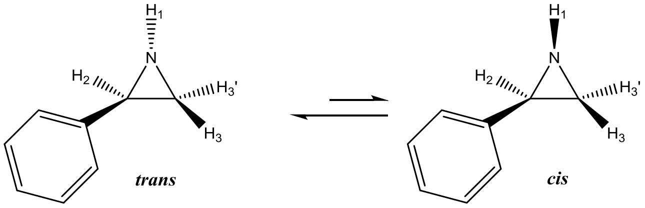

298K

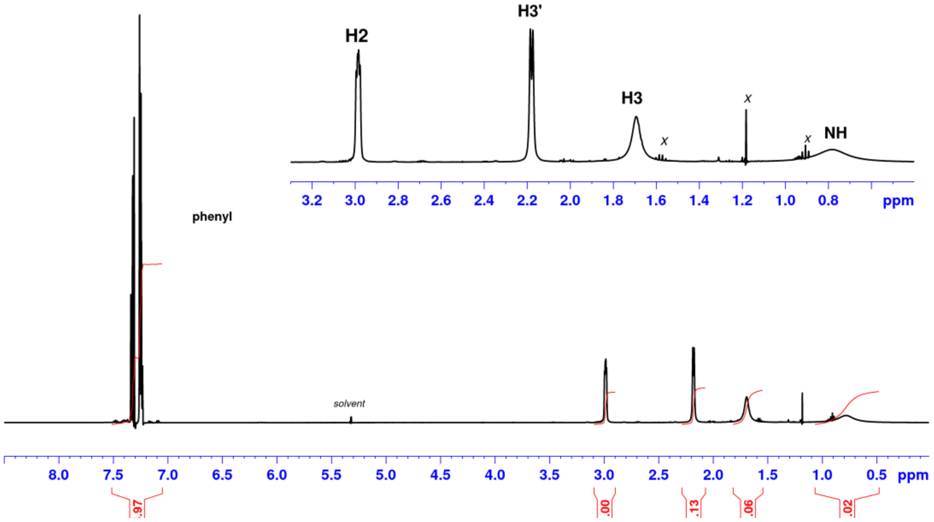

193K

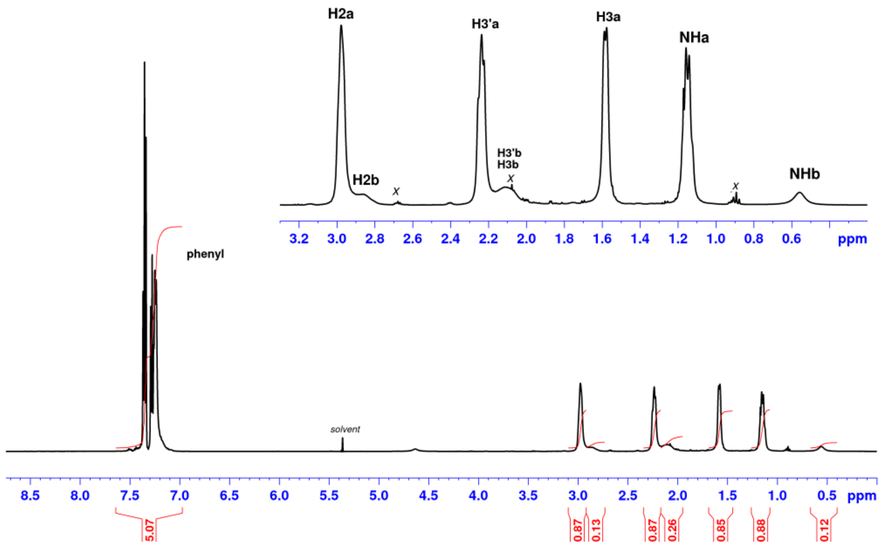

Figure S2: NOESY Spectrum of 2-phenylaziridine (**1a**) in CD_2Cl_2 at 298 K (500 MHz). The anomalous cross-peaks showing the same sign as the diagonal reference peaks are clearly seen (red), whilst conventional dipolar NOEs are observed to have opposite sign to the diagonal-peaks (blue). The weak positive cross-peak between H2 and H3 also has its origins in scalar relaxation of the first kind since the H2-H3 scalar coupling is modulated by the nitrogen inversion (see Fig. S7). The weak (dispersive) anti-phase peaks between H2 and H3' arise from zero-quantum coherences that were not suppressed in the NOESY sequences employed.

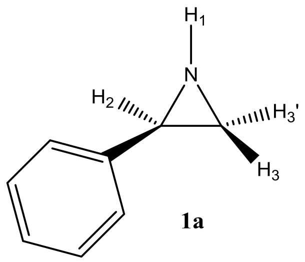

1a

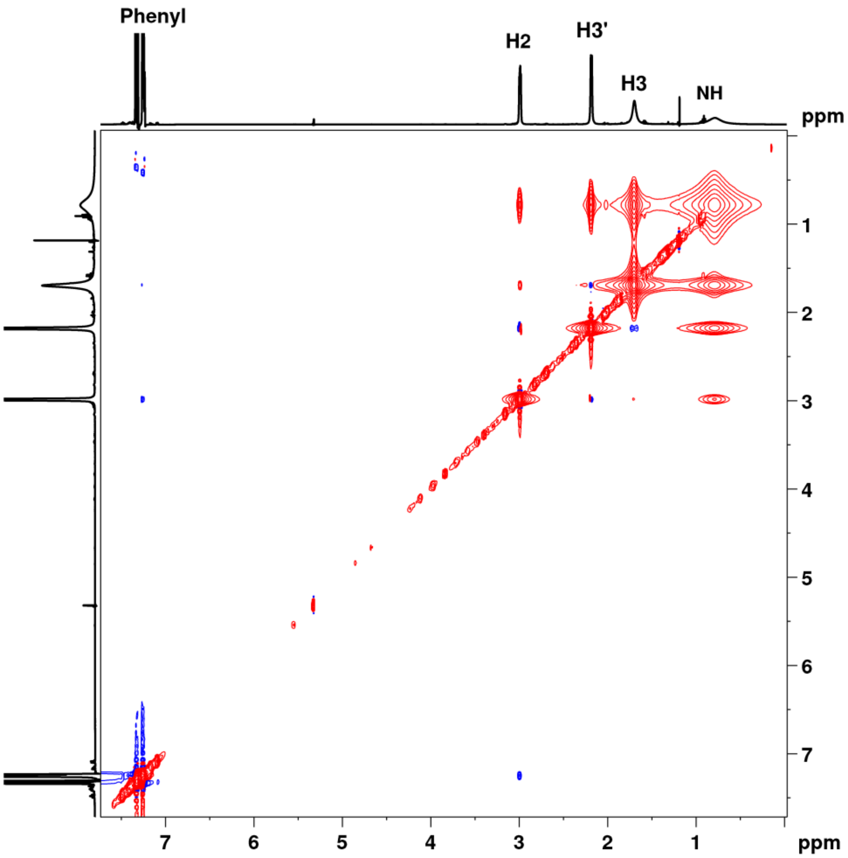

Figure S3: NOESY Spectrum of 2-(chlorophenyl)aziridine (**1b**) in C_6D_6 at 298 K (500 MHz). The anomalous peaks due to scalar cross-relaxation are again apparent with positive sign (red) in addition to the dipolar NOEs (blue).

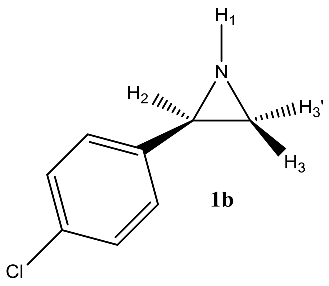

1b

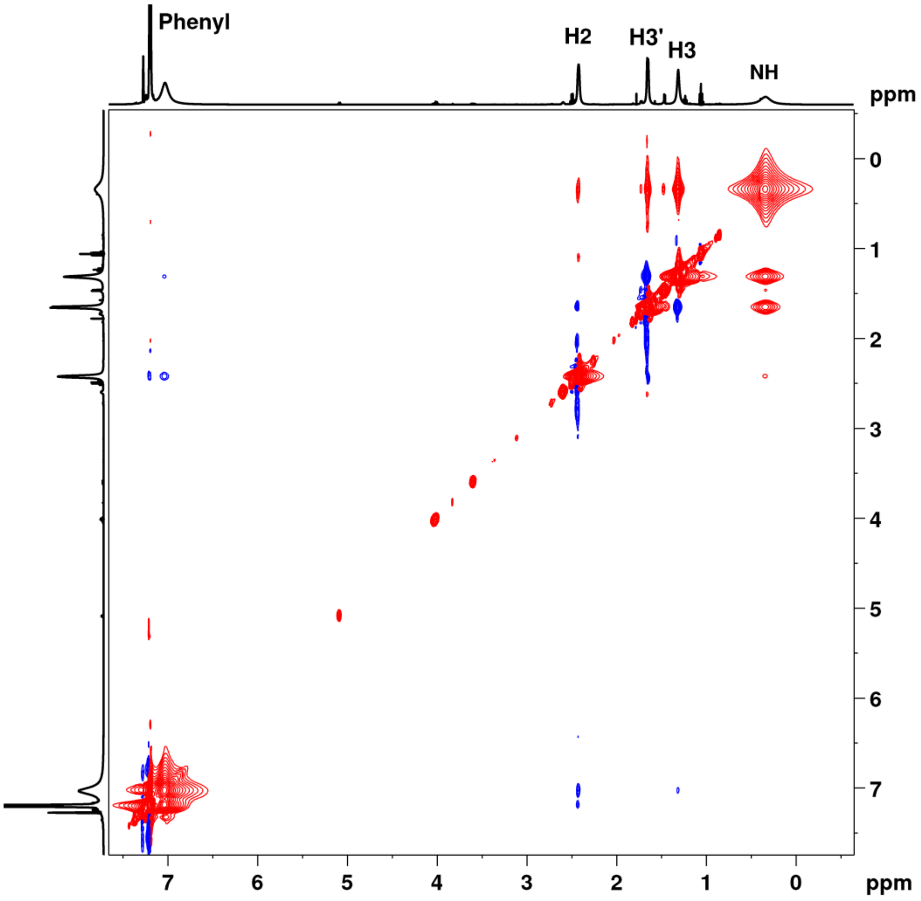

Figure S4: NOESY Spectrum of 2-methylaziridine (**1c**) in CD_2Cl_2 at 298 K (500 MHz). The anomalous peaks due to scalar cross-relaxation are again apparent with positive sign (red).

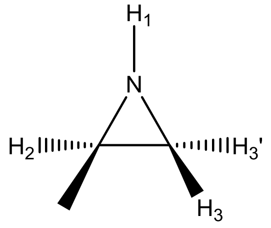

1c

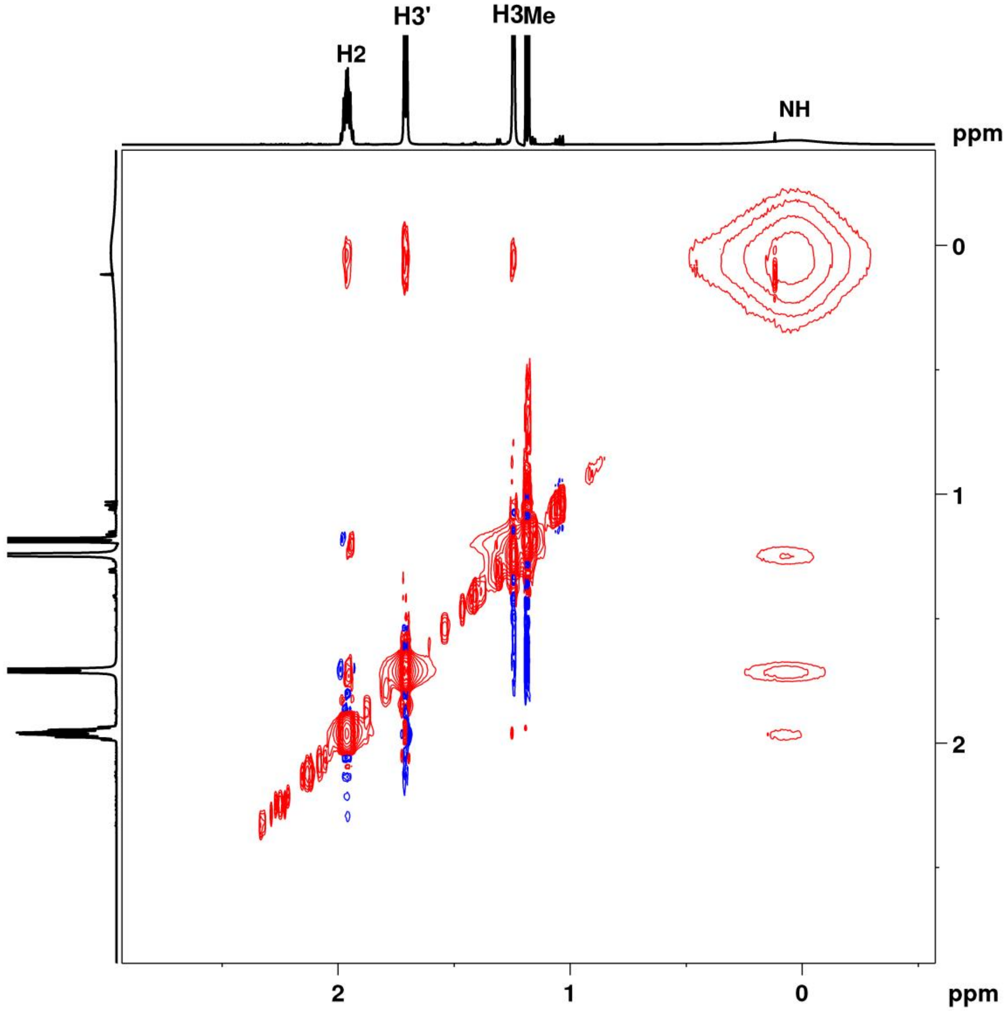

Figure S5: NOESY Spectrum of 2,2-dimethylaziridine (**1d**) in CD_2Cl_2 at 298 K (500 MHz). The anomalous peaks due to scalar cross-relaxation are again apparent with positive sign (red) between the NH and the scalar coupled H3, H3’ protons; no such peaks exist to the methyl protons.

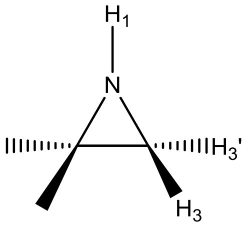

1d

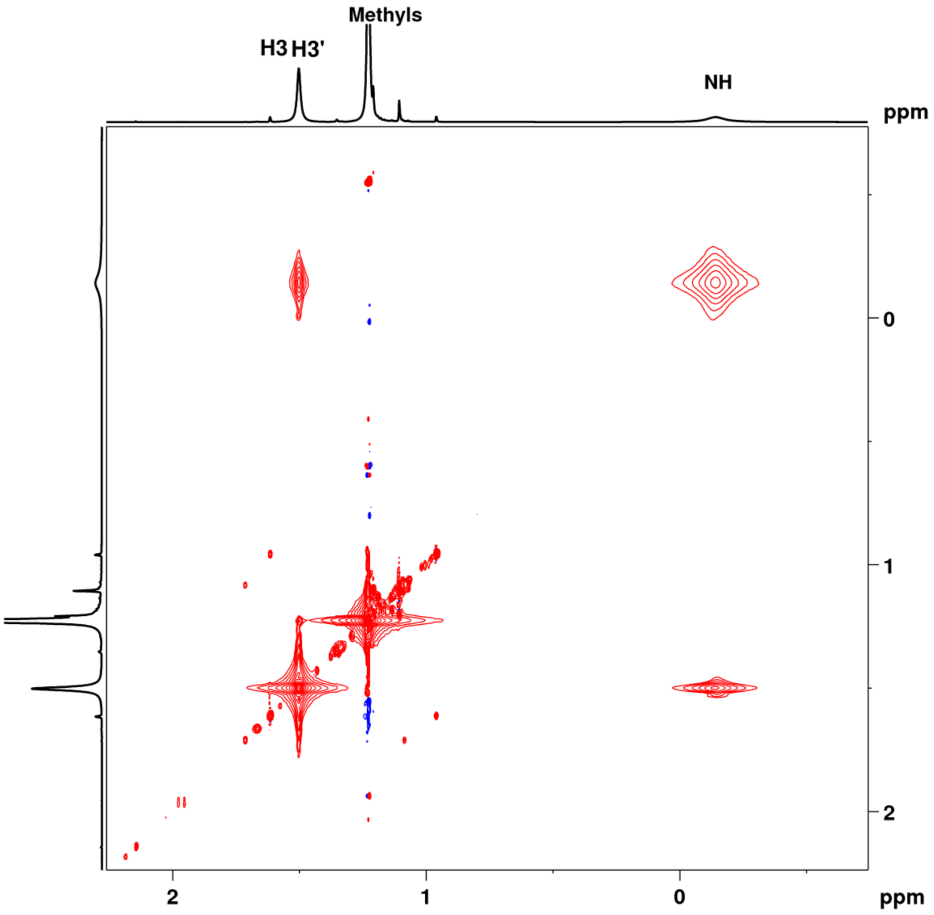

Figure S6: NOESY spectrum of the isotopically enriched ^{15}N-2-phenylaziridine (**1a**) in CD_2Cl_2 at 298 K (500 MHz). The anomalous cross-peaks are still clearly seen and thereby the involvement of the quadrupolar ^{14}N nucleus in the origin of these peaks is excluded.

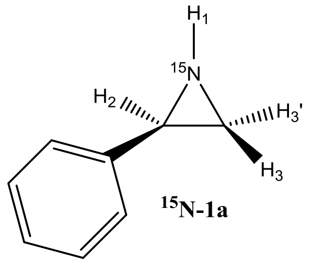

^{15}N-1a

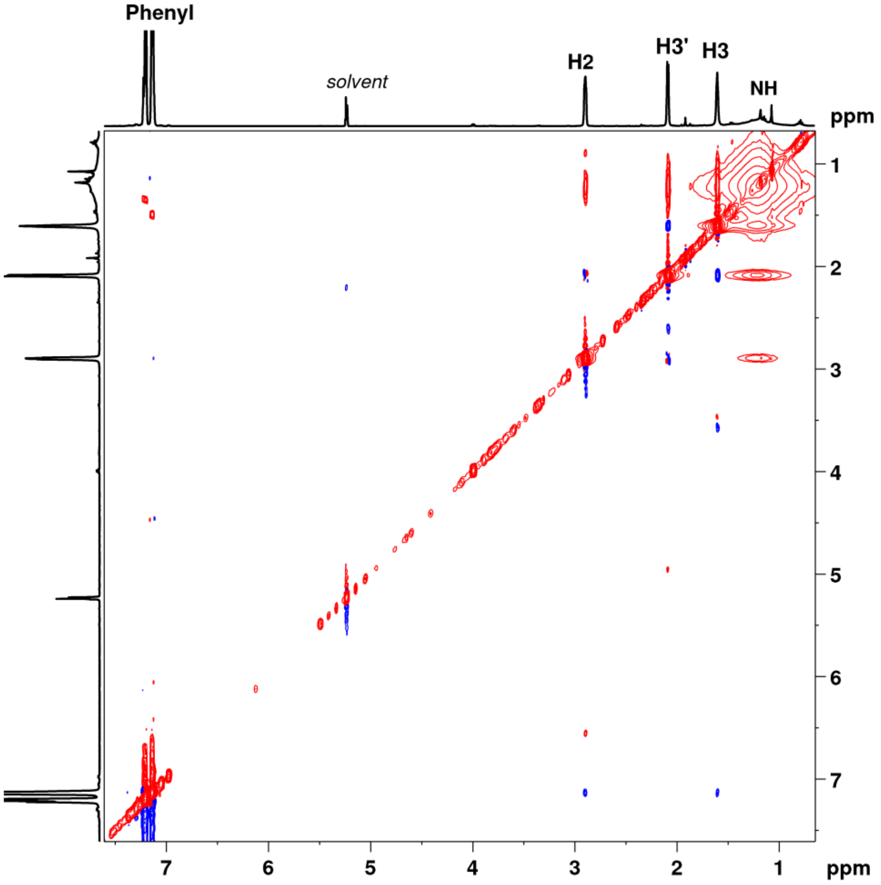

Figure S7: Modulation of vicinal and geminal ^{1}H-^{1}H *J*-couplings not involving the amine proton during aziridine nitrogen centre inversion process, computed as described in the caption to Figure 2 in the main text.

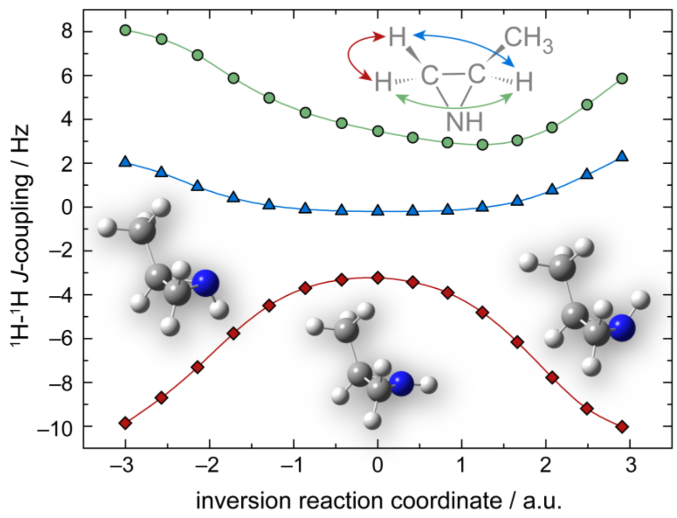

Figure S8: An illustration to the fact that the SRFK process is essential for the correct description of the cross-relaxation in substituted aziridines. (A) Theoretical NOESY spectrum of 2-methylaziridine (**1c**) computed as described in the main text. (B) Theoretical NOESY spectrum computed with SRFK contributions to the relaxation superoperator excluded from the simulation.

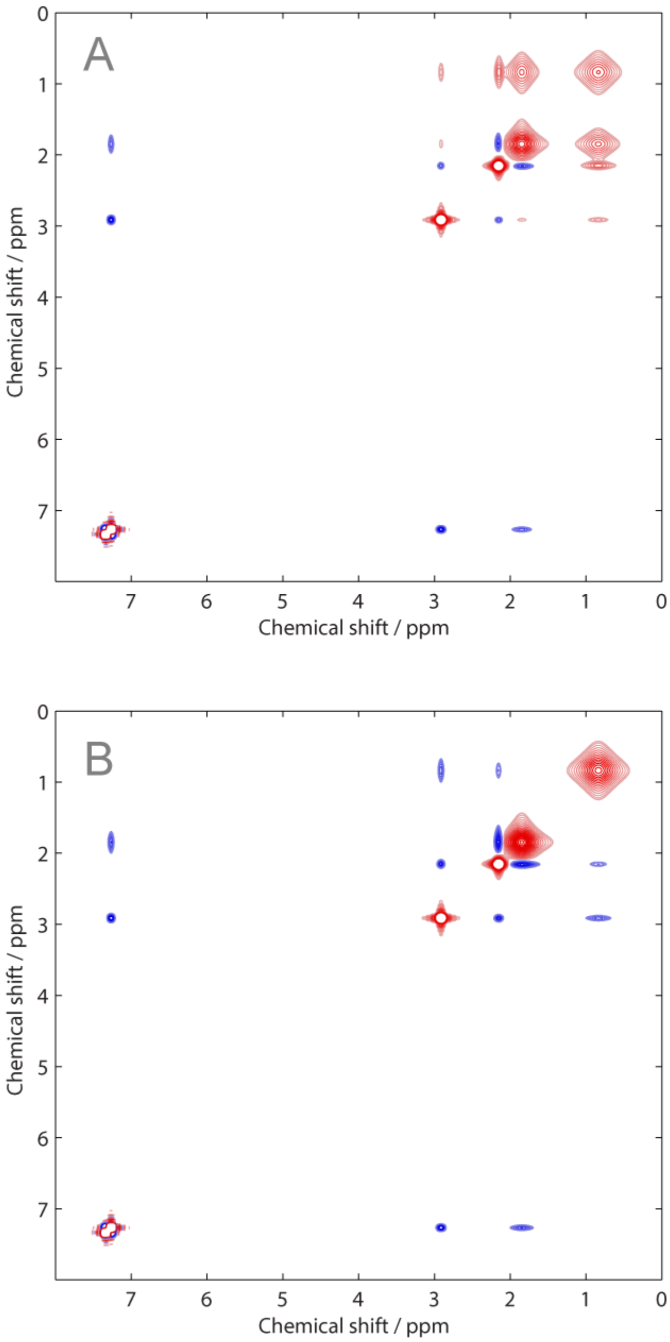

Figure S9: NOESY Spectra of azetidine (**2**) and pyrrolidine (**3**) in $CDCl_3$ at 298 K (500 MHz). In both cases positive scalar cross-relaxation NOESY cross-peaks are not apparent and the NH resonances remain sharp (scalar coupling interferences are apparent in the NOE cross-peaks between CH protons; these NOEs would be expected to be weak in these very small molecules).

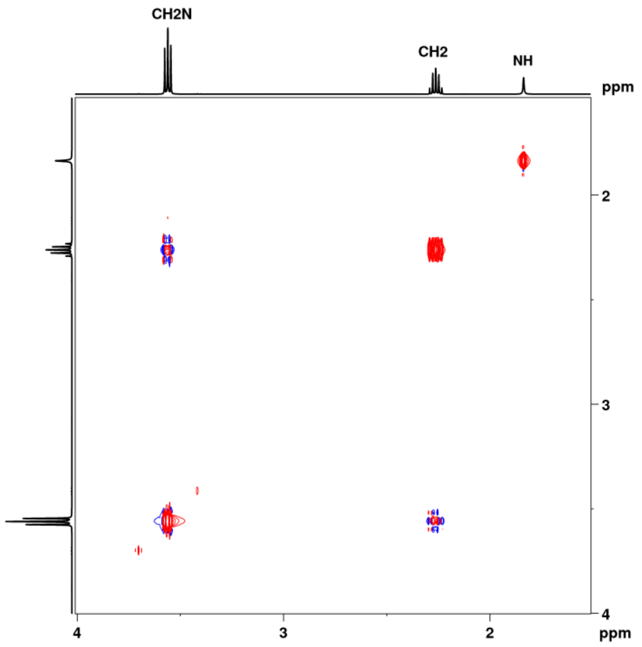

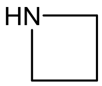

2

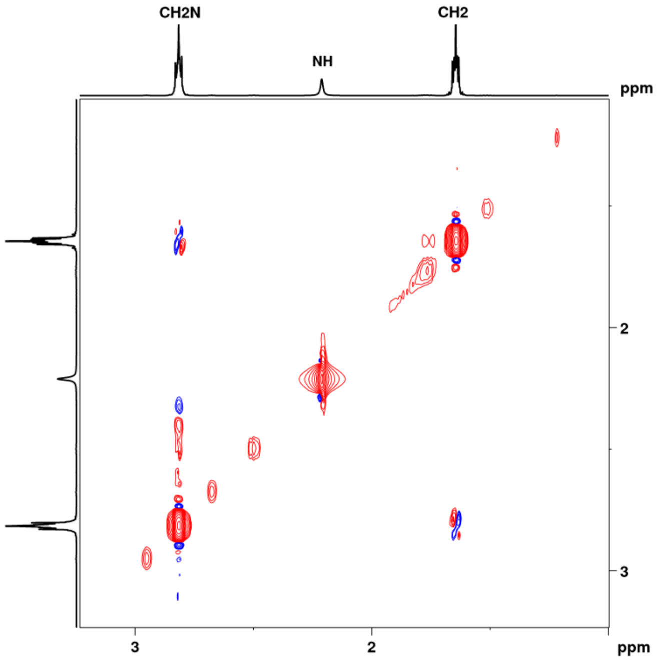

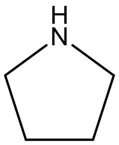

3

References

[1] a) S. Gabriel, J. Colman, *Ber. Dtsch. Chem. Ges.* **1914**, *47*, 1866-1873; b) F. Wolfheim, *Ber. Dtsch. Chem. Ges.* **1914**, *47*, 1440-1452.

[2] B. R. Buckley, A. P. Patel, K. G. U. Wijayantha, *J. Org. Chem.* **2013**, *78*, 1289-1292.

[3] X.-Y. Dou, L.-N. He, Z. Z. Yang, J.-L. Wang, *Synlett* **2010**, 2159-2163.

[4] R. Wrigglesworth, C. S. J. Walpole, S. J. Bevan, E. A. Campbell, A. Dray, G. A. Hughes, I. James, K. J. Masdin, J. J. Winter, *J. Med. Chem.* **1996**, *39*, 4942-4951.

[5] G. Lamaty, J.-Y. Sanchez, A. Sivade, J. Wylde, *J. Bull. Soc. Chim. Fr* **1985**, 1261-1266.

[6] H. Yamanaka, J. Matsuo, A. Kawana, T. Mukaiyama, *ARKIVOC* **2004**, *3*, 42-65.

[7] Y. Minoura, M. Takebayashi, C. C. Price, *J. Am. Chem. Soc.* **1959**, *81*, 4689-4691.

[8] X. Li, N. Chen, J. Xu, *Synthesis* **2010**, 3423-3428.

[9] B. R. Friedman, J. P. Chauvel, N. S. True, *J. Am. Chem. Soc.* **1984**, *106*, 7638-7639.

[10] H. J. Hogben, M. Krzystyniak, G. T. P. Charnock, P. J. Hore, I. Kuprov, *J. Magn. Reson.* **2011**, *208*, 179-194.

[11] a) R. R. Ernst, G. Bodenhausen, A. Wokaun, *Principles of nuclear magnetic resonance in one and two dimensions, Vol. 14*, Oxford University Press, Oxford, **1987**; b) A. G. Redfield, *IBM J. Res. Dev.* **1957**, *1*, 19-31.

[12] M. J. T. Frisch, G. W.; Schlegel, H. B.; Scuseria, G. E.; Robb, M. A.; Cheeseman, J. R.; Scalmani, G.; Barone, V.; Mennucci, B.; Petersson, G. A.; Nakatsuji, H.; Caricato, M.; Li, X.; Hratchian, H. P.; Izmaylov, A. F.; Bloino, J.; Zheng, G.; Sonnenberg, J. L.; Hada, M.; Ehara, M.; Toyota, K.; Fukuda, R.; Hasegawa, J.; Ishida, M.; Nakajima, T.; Honda, Y.; Kitao, O.; Nakai, H.; Vreven, T.; Montgomery, J. A., Jr.; Peralta, J. E.; Ogliaro, F.; Bearpark, M.; Heyd, J. J.; Brothers, E.; Kudin, K. N.; Staroverov, V. N.; Kobayashi, R.; Normand, J.; Raghavachari, K.; Rendell, A.; Burant, J. C.; Iyengar, S. S.; Tomasi, J.; Cossi, M.; Rega, N.; Millam, N. J.; Klene, M.; Knox, J. E.; Cross, J. B.; Bakken, V.; Adamo, C.; Jaramillo, J.; Gomperts, R.; Stratmann, R. E.; Yazyev, O.; Austin, A. J.; Cammi, R.; Pomelli, C.; Ochterski, J. W.; Martin, R. L.; Morokuma, K.; Zakrzewski, V. G.; Voth, G. A.; Salvador, P.; Dannenberg, J. J.; Dapprich, S.; Daniels, A. D.; Farkas, Ö.; Foresman, J. B.; Ortiz, J. V.; Cioslowski, J.; Fox, D. J., Gaussian, Inc., Wallingford CT, **2009**.

[13] a) A. Abragam, *The principles of nuclear magnetism*, Clarendon Press, Oxford, **1961**; b) S. Szymanski, G. Binsch, *J. Magn. Reson. (1969)* **1990**, *87*, 166-168; c) L. G. Werbelow, J. Kowalewski, *J. Chem. Phys.* **1997**, *107*, 2775-2781; dN. C. Pyper, *Mol. Phys.* **1971**, *21*, 961-&.

[14] a) M. Goldman, *J. Magn. Reson.* **1984**, *60*, 437-452; b) A. Kumar, R. C. R. Grace, P. K. Madhu, *Prog. Nucl. Magn. Reson. Spectrosc.* **2000**, *37*, 191-319.

[15] a) I. Kuprov, *J. Magn. Reson.* **2011**, *209*, 31-38; b) I. Kuprov, N. Wagner-Rundell, P. J. Hore, *J. Magn. Reson.* **2007**, *184*, 196-206.

[16] D. Neuhaus, M. P. Williamson, *The nuclear Overhauser effect in structural and conformational analysis*, 2nd ed., Wiley, New York, **2000**.

[17] a) M. Eisenstadt, A. G. Redfield, *Phys. Rev.* **1963**, *132*, 635-643; b) L. P. Hwang, J. H. Freed, *J. Chem. Phys.* **1975**, *63*, 4017-4025; c) H. C. Torrey, *Phys. Rev.* **1953**, *92*, 962-969.

[18] Y. Xue, I. S. Podkorytov, D. K. Rao, N. Benjamin, H. L. Sun, N. R. Skrynnikov, *Protein Sci.* **2009**, *18*, 1401-1424.

[19] M. Goldman, *J. Magn. Reson.* **2001**, *149*, 160-187.
